# Transcriptomic Profiling Revealed Plexin A2 Downregulation With Migration and Invasion Alteration in Dacarbazine-Treated Primary Melanoma Cells

**DOI:** 10.3389/fonc.2021.732501

**Published:** 2021-12-03

**Authors:** Anna Tyumentseva, Anton Averchuk, Nadezhda Palkina, Ivan Zinchenko, Anton Moshev, Andrey Savchenko, Tatiana Ruksha

**Affiliations:** ^1^ Department of Pathophysiology, Krasnoyarsk State Medical University, Krasnoyarsk, Russia; ^2^ Federal Research Center Krasnoyarsk Science Center of the Siberian Branch of the Russian Academy of Sciences, Krasnoyarsk, Russia; ^3^ Laboratory of Cell Molecular Physiology and Pathology, Federal Research Center, Krasnoyarsk Science Center of The Siberian Branch of The Russian Academy of Sciences, Krasnoyarsk, Russia

**Keywords:** migration, transcriptome, dacarbazine, PLNXA2, melanoma

## Abstract

Melanoma is highly heterogeneous type of malignant neoplasm that is responsible for the majority of deaths among other types of skin cancer. In the present study, we screened a list of differentially expressed genes in two primary, drug-naïve melanoma cell lines derived from patients with melanoma following treatment of the cells with the chemotherapeutic agent dacarbazine. The aim was to determine the transcriptomic profiles and associated alterations in the cell phenotype. We found the vascular endothelial growth factor A/vascular endothelial growth factor receptor 2, phosphoinositide 3-kinase/protein kinase B and focal adhesion signaling pathways to be top altered after dacarbazine treatment. In addition, we observed the expression levels of genes associated with tumor dissemination, integrin β8 and matrix metalloproteinase-1, to be diminished in both cell lines studied, the results of which were confirmed by reverse transcription-quantitative polymerase chain reaction. By contrast, plexin A2 expression was found to be upregulated in K2303 cells, where reduced migration and invasion were also observed, following dacarbazine treatment. Plexin A2 downregulation was associated with the promotion of migrative and invasive capacities in B0404 melanoma cells. Since plexin A2 is semaphorin co-receptor that is involved in focal adhesion and cell migration regulation, the present study suggested that plexin A2 may be implicated in the dacarbazine-mediated phenotypic shift of melanoma cells. We propose that the signature of cancer cell invasiveness can be revealed by using a combination of transcriptomic and functional approaches, which should be applied in the development of personalized therapeutic strategies for each patient with melanoma.

## Introduction

Melanoma is a highly heterogeneous type of tumor in which surgical excision in the early stages of the disease confers high survival rates (1, 2). However, various treatment options, including targeted therapy, immunotherapy and chemotherapy, are not completely effective against advanced melanoma (3). Dacarbazine (DTIC) is a chemotherapeutic agent that is used as a form of monotherapy or combined chemotherapy for melanoma (4, 5). However, the overall response rate to DTIC rarely exceeds 17.6% (4, 5). Mechanistically, DTIC is an alkylating agent that promotes G_1_-phase cell cycle arrest (6). Alkylating agents favor the formation of O6-alkylguanine derivatives, which bind to thymine instead of cytosine during DNA replication, thereby blocking cell division and causing cell death at the G_1_ and S phases (7).

Drug resistance to alkylating agents develops in cancer cells through a number of mechanisms. Induction of tumor heterogeneity can be considered one such mechanism of this process, which results in genetic differences among different cell types within the tumor, in addition to the acquisition of epigenetic alterations by cancer cells during tumorigenesis (8). Indeed, a previous transcriptomic study revealed >70 differentially expressed genes between temozolomide-sensitive and temozolomide-resistant glioma cells (9). In addition, metabolic heterogeneity can give rise to differences in responses by cancer cells to oxidative stress, lactate uptake and pyruvate metabolism (10). It has been previously reported that cancer cells with metastatic phenotypes tend to exhibit enhanced rates of lactate transport, whilst oxidative stress suppresses metastasis (11). Various types of tumors have demonstrated the ability to create pro-tumorigenic hypoxic environments, such that they can create physical barriers by altering blood flow to reduce drug exposure (12). Pancreatic cancer cells provide hypoxia-inducible factor 1α-mediated sonic hedgehog ligand secretion to form fibrous tissue deposition causing a poor drug delivery (13). Non-small-cell lung cancer cells release increased amounts of lactate to potentiate hypoxic-acidic microenvironment and to suppress cytotoxic T-cell activation (14). Drug resistance of melanoma cells to DTIC was reported to be mediated by hypoxia through activation of the nodal signaling pathway (15). The protein kinase B (AKT)/mechanistic target of rapamycin (mTOR) signaling pathway was shown to be up-regulated in DTIC-treated T24.6.9 melanoma cells (16). Furthermore, insulin can weaken the sensitivity of melanoma cells to DTIC by triggering the phosphoinositide 3-kinase (PI3K)/mTOR signaling pathway (17). Therefore, manipulations to various molecular signaling pathways may be underlying the diminished efficacy of chemotherapeutic agents that operate by alkylation.

In this study, we analyzed the effect of DTIC on the melanoma transcriptomic profile. Specific focus was placed on the signal pathways that can regulate cell migration, invasion and adhesion, all of which are associated with shifts in their metastatic phenotypes.

## Materials And Methods

### Tissue Samples and Cell Culture

The present study was approved by the Ethics Committee of the Krasnoyarsk State Medical University (protocol no. 73/2016; approval date, 16 December, 2016) and the Krasnoyarsk Regional Clinical Oncology Center named after A.I. Kryzhanovskiy (protocol no. 8, 14, June, 2017). All procedures were performed according to the Institutional Safety Instructions, which included biosecurity. All researchers are trained in Institutional Safety/Biosafety and follow-up instructions are provided periodically every 3 months, followed by signature confirmation. The present study was also approved by the Institutional Bioethical Commission (protocol no. 3; approval date, 27 October 2017).

The operative samples were obtained from the Department of General Oncosurgery of Krasnoyarsk Regional Clinical Oncology Center named after A.I. Kryzhanovskiy and primary cell lines were prepared as described previously (18). In the present study, two primary melanoma cell populations were obtained from two drug-naïve melanoma patients. Written informed consent was obtained from each patient before the study. Inclusion criteria were as follows: pathologically confirmed primary malignant melanoma and no know second primary malignancies; age more than 18 years old; no systemic treatment in the four weeks prior to surgical treatment. Tumor tissues predominantly consisted of tumor cells that did not contain an abundance of stromal or vascular cell types. They were processed immediately after surgical excision and melanoma cell suspensions were prepared within 3 h. Single cell suspensions were obtained by mechanical dissociation of tumor tissues. These two melanoma cell populations were named K2303 and B0404 thereafter.

K2303 cells were obtained from a 30-year-old female patient with the melanoma localized on the right leg. It exhibited a superficial type of spreading with a Breslow’s depth of 2.2 mm. B0404 cells were obtained from a 55-year-old male patient, the melanoma was localized on the right shin with a superficial spreading type and Breslow’s depth of 2.0 mm.

Cells were cultured in RPMI-1640 medium with L-glutamine (Gibco, Thermo Fisher Scientific, Inc.) and 10% fetal bovine serum (FBS; Gibco, Thermo Fisher Scientific, Inc.) in the presence of antibiotics and antimycotics (10,000 U/ml penicillin G, 10,000 µg/ml streptomycin and 25 µg/ml amphotericin B; HyClone; Cytiva) at 37°C and 5% CO_2_ using a CO_2_ incubator (Sanyo Electric Co., Ltd.).

### DTIC Treatment and Cell Viability Evaluation

To estimate the 50% inhibitory concentration (IC_50_) of DTIC, cells were treated with a series of DTIC (Sigma-Aldrich Co., USA) dilutions. For this purpose, melanoma cells were placed in a 96-well plate at a density of 1x10^4^ cells per ml and cultured for 48 h at 37°С. In total, four different concentrations of DTIC in DMSO (Panreac Quimica S.A.) were added to the culture medium to achieve the final concentrations of 250, 500, 750 and 1,000 mg/l. Since each dose also contains 1% DMSO, 1% DMSO was used as the negative control. The cells were incubated with DTIC for 72 h at 37°С and 5% CO_2_.

To evaluate cell viability, 3-(4,5-dimethylthiazol-2-yl)-2,5-diphenyltetrazolium bromide (MTT; Invitrogen; Thermo Fisher Scientific, Inc.) assay was used. The culture medium was first replaced with that without the drug before 0.5 mg/ml MTT was added. After 4 h at 37°С the cells were lysed with DMSO and the amount of formazan formed was evaluated using an EFOS-9305 spectrophotometer (Shvabe Photosystems, Jsc) at a wavelength of 560 nm to measure light absorbance. The experiment was performed in triplicate. Using the dose-dependency curve of DTIC-mediated inhibition, IC_50_ was calculated using the GraphPad Software Prism 7.05/e (San Diego, CA, USA).

### Evaluation of Apoptosis in Melanoma Cells After DTIC Treatment

Melanoma cell lines were seeded into 24-well plates at a density of 1x10^5^ cells per ml. After 24 h the cells were treated with 5.5 mM DTIC or 1% DMSO. In parallel, 1% DMSO was used as the negative control. After 72 h at 37°C and 5% CO_2_ incubation the cells were stained using an annexin V-fluorescein isothiocyanate (FITC)/7-aminoactinomycin D (7-AAD) kit (Immunotech; Beckman Coulter, Inc.) according to the manufacturer’s protocol. For this, 10 μl of Annexin V-FITC and 20 μl of 7-AAD were added to each sample containing 100 μl of melanoma cell suspension in binding buffer at a concentration of 5x10^6^, incubated for 15 min in the dark on ice, then 400 μl of binding buffer was added followed by analysis using a Cytomics FC-500 flow cytometer (Beckman Coulter Inc.) with NAVIOS software v.1.3. (Beckman Coulter, Brea, USA).

After detection, with the distribution of cells displayed in their respective regions of two-parameter diagrams, living cells were defined as unstained. By contrast, cells were defined to be in early apoptosis if they stained positive for phosphatidylserine by annexin V-FITC but negative for 7-AAD due to preserved membrane integrity. Cells were defined to be at late-stage apoptosis and necrotic if there is evidence of violation in the integrity of the membranes and were stained simultaneously with both fluorescent dyes. Cells that stained positive for annexin V alone were defined as necrotic cells. The experiment was performed in triplicate.

### Migration and Invasion Assay of Melanoma Cells Under DTIC Treatment

Melanoma cells were plated into six-well plates at a density of 1x10^5^ cells per ml. After 24 h at 37°C and 5% CO_2_, the cells were treated with 5.5 mM DTIC or 1% DMSO. After 72 h, the cells were transferred to serum-free RPMI-1640 medium with 5.5 mM DTIC and diluted to a final density of 1x10^4^ cells per ml. Cell migration and invasion assays were performed using CytoSelect^™^ 24-Well Cell Migration and Invasion assays (8 μm, colorimetric format; Cell Biolabs, Inc.) according to the manufacturer’s protocols. Briefly, a 300 μl suspension of melanoma cells in RPMI-1640 medium without serum was placed into the upper part of special chambers for migration and invasion. The bottom layer of the chambers for migration assays was made of a polycarbonate membrane with pores, whilst for measuring invasion it was lined additionally with a layer of Matrigel. The chambers containing the cell suspension were incubated in a CO_2_ incubator at 37°C and 5% CO_2_ for 24 h in the wells of a 24-well plate filled with RPMI-1640 medium containing 10% FBS, which constitutes the lower part of the chamber. After incubation, the chambers were removed and the Matrigel was mechanically removed. Cells that did not migrate or invade located on the membrane inside the chamber and the invasive and migratory cells located on the outer side of the membrane were fixed and, stained by Cell Stain Solution, washed in dH20, dried at room temperature for 10 min and then lysed by Extraction Solution. The color intensity levels of lysates were measured on an EFOS-9305 spectrophotometer at a wavelength 560 nm, which were used to calculate the levels of cell migration and invasion. The experiment was performed in triplicate.

### Whole Transcriptome Assay

Total RNA was extracted using RecoverAll™ Total Nucleic Acid Isolation kit (Invitrogen™; Thermo Fisher Scientific, Inc.) according to the manufacturer’s protocol, using on-column DNase digestion. Extracted RNA was examined using a Qubit^®^ 2.0 fluorimeter (Invitrogen; Thermo Fisher Scientific, Inc.) with the use of a Qubit^®^ RNA HS Assay kit (Invitrogen by Thermo Fisher Scientific, Inc.). In total, 10 ng total RNA was amplified, purified, reverse transcribed and labeled with biotin using the GeneChip™ WT Plus kit (cat # 902280, Affymetrix; Thermo Fisher Scientific, Inc.) following the manufacturer’s protocols. The samples were then hybridized to GeneChip™ HuGene 2.1 ST Array Strips (cat # 902114, Applied Biosystems; Thermo Fisher Scientific, Inc.). Post-hybridization staining and washing were processed according to manufacturer’s protocol using GeneAtlas™ Hybridization, Wash, and Stain kit for WT array strips (cat # 900720-C, Affymetrix; Thermo Fisher Scientific, Inc.). The strips were scanned in a GeneAtlas™ Imaging Station (Affymetrix; Thermo Fisher Scientific, Inc.). Data were collected using the Transcriptome Analysis Console software version 4.0.0 and subsequent releases (Thermo Fisher Scientific, Inc.).

A total of three replicates of each cell line before and after DTIC treatment at 5.5 mM for 72 h were prepared. Quality Control of the experiment was automatically estimated at the imaging stage and all arrays passed the quality controls. The data generated in the present study were deposited at the Array Express repository (accession no. E-MTAB-10359; https://www.ebi.ac.uk/arrayexpress/experiments/E-MTAB-10359/).

The expression data were used to perform principal components analysis using all probe sets by Transcriptome Analysis Console Software v.4.0.1. (Thermo Fisher Scientific, Inc.). The expression data were also used to cluster the samples using a hierarchical clustering method (19). All P-values were false discovery rate-corrected for multiple hypothesis testing. Differentially expressed probe sets were defined using the threshold of absolute fold change ≥2 and the Q-value ≤0.05. PANTHER™ v.10.0 classification system (URL: PanterDB.org) was used to interpret the biological function of the differentially expressed genes.

### RNA Isolation and Reverse Transcription-Quantitative Polymerase Chain Reaction (RT-qPCR) for Gene Expression Studies

The cells were seeded at a density of 2x10^5^ cells per ml and cultured for 24 h at 37°C and 5% CO_2_. The culture medium was then replaced and the cells were treated with DTIC at a final concentration of 5.5 mM, with 1% DMSO used as the negative control. The cells were then incubated for 72 h, detached with 0.25% trypsin-ethylenediaminetetraacetic acid and washed twice with cold 0.01 M phosphate buffered saline (Amresco LLC). Isolation of total RNA was performed using the RecoverAll™ Total Nucleic Acid Isolation kit (Ambion; Thermo Fisher Scientific, Inc.) according to the manufacturer’s protocol. Total RNA was then subjected to reverse transcription with random primers using the MMLV RT kit (cat # SK021, Evrogen, Russia). The reaction was performed with incubation at 40°C for 50 min, and the enzyme was subsequently inactivated by incubation at 70°C for 10 min.

Amplification of the obtained cDNA in an amount of 2 μl was performed in a StepOne ™ Real-Time PCR System (Applied Biosystems; Thermo Fisher Scientific, Inc.) in 20 μl reaction mixture containing 1 μl probe and primers for the detection of integrin β8 (ITGB8), plexin-A2 (PLXNA2) and matrix metalloproteinase-1 (MMP-1) from the TaqMan™ Gene Expression Assay (Assay names, CYP1A1 Hs01054796_g1, CYP1A2 Hs00167927_ml, CYP2E1 Hs00559367_ml, ITGB8 Hs00174456_ml, PLXNA2 Hs00300697_ml and MMP1 Hs00899658_ml; cat. no. 4331182; Applied Biosystems, Thermo Fisher Scientific Inc.) and 8 μl 2.5X reaction mixture for RT-PCR (Syntol, Russia) in the presence of the reference ROX dye (Syntol, Russia), using the following thermocycling protocol: 50°C for 2 min and 95°C for 10 min, followed by 40 cycles of denaturation at 95°С for 15 sec and annealing and elongation at 60°С for 1 min. Endogenous normalizing controls were β-actin and hypoxanthine phosphoribosyltransferase-1 (Assay names, ACTB Hs01060665_g1 and HPRT1 Hs01003267_m1; TaqMan™ Gene Expression Assay; cat. no. 4331182; Applied Biosystems; Thermo Fisher Scientific, Inc.). Relative expression levels were calculated using the 2^-ΔΔCq^ method as previously described (20).

### Statistical Analysis

Statistical analysis was performed using the non-parametric Mann-Whitney U-test for comparisons between two independent groups (DTIC-treated group vs. control untreated group) using the Statistica 6.1 software (StatSoft, Inc.). P<0.05 was considered to indicate a statistically significant difference.

## Results

### Sensitivity of Melanoma Cells Derived From Different Patients to DTIC and CYP1A1 Expression

**Figure 1:**
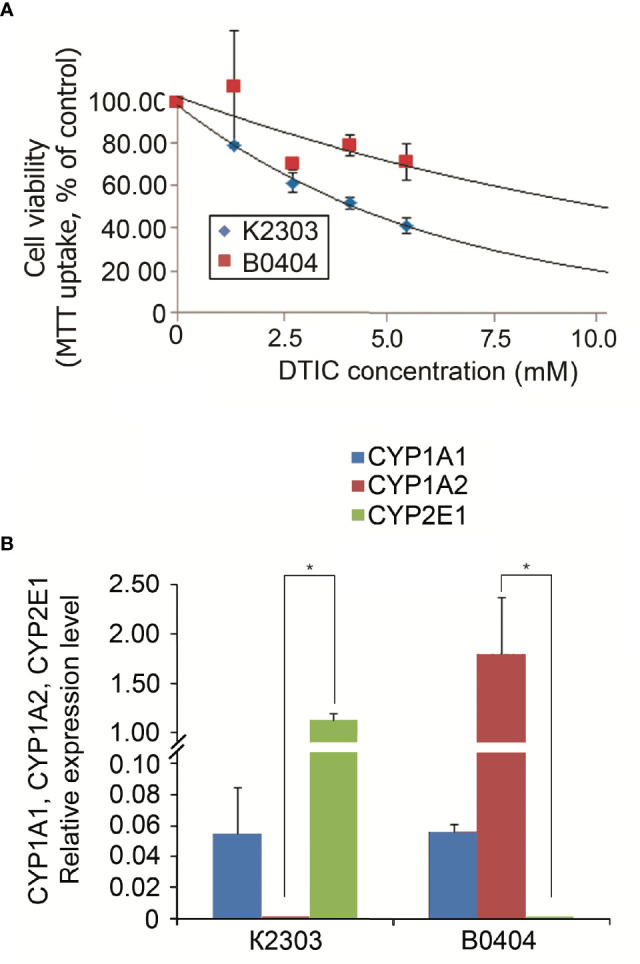
**(A)** Sensitivity of melanoma cell lines K2303 and B0404 to dacarbazine. 3-(4,5-dimethylthiazol-2-yl)-2,5-diphenyltetrazolium bromide-assay-based dose-response curves were used to estimate the 50% inhibitory concentration. IC_50_ was calculated using the GraphPad Software Prism 7.05/e Data are presented as the mean ± SEM. **(B)** Relative *CYP1A1*, *CYP1A2* and *CYP2E1* gene expression levels according to reverse transcription-quantitative polymerase chain reaction in K2303 and B0404 melanoma cells. Data are presented as the mean ± SEM. ^*^P<0.05 by Mann-Whitney U test for unpaired samples.

According to MTT analysis, the IC_50_ value of DTIC for the K2303 melanoma cells was calculated to 4.2 mM, whilst the IC50 of DTIC for B0404 melanoma cells equated to 10.3 mM (**Figure 1A**). The final concentration of DTIC was chosen to be 5.5 mM. Overall, K2303 melanoma cells were found to be more sensitive to DTIC treatment compared with B0404 cells. As DTIC metabolized extrahepatically by cytochrome P450 1A1 (CYP1A1), it’s mRNA levels were estimated in K2303 and B0404 cells by qRT-PCR as well as CYP1A2 and CYP2E1 levels which can metabolize DTIC also (21). We determined CYP1A1 expression in both cell lines studied, CYP1A2 expression in B0404 and CYP2E1 expression in K2303 cells (**Figure 1** **В**). Relative expression level was.

### DTIC Induces Apoptosis in Primary Melanoma Cells

**Figure 2:**
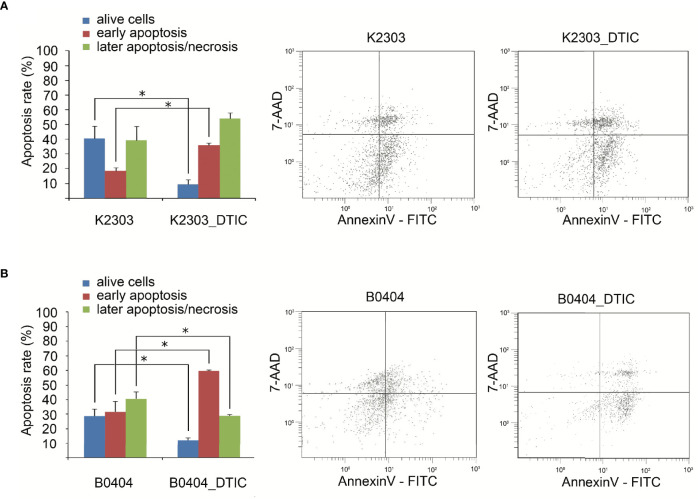
Effect of dacarbazine at 5.5 mM on the apoptosis of melanoma cells according to the results of the annexin V/7-aminoactomycin flow cytometry assay. **(A)** The percentages of live, early-apoptotic, late-apoptotic and necrotic cells are shown in the two-parameter scatterplots for K2303 melanoma cells. **(B)** The percentages of live, early-apoptotic, late-apoptotic and necrotic cells are shown in the two-parameter scatterplots for B0404 melanoma cells. Data are presented as the mean ± SEM. ^*^P<0.05 by Mann-Whitney U test for unpaired samples.

To evaluate the sensitivity of the primary melanoma cells originated from the two patients to DTIC *in vitro*, the proportions of live and apoptotic cells were measured using flow cytometry before and after DTIC treatment. Both cell lines demonstrated notable responses to treatment, since DTIC increased the percentage of cells in early apoptosis by two-fold (**Figure 2**). The proportion of late apoptotic and necrotic cells was not altered in K2303 and reduced in B0404.

### DTIC Treatment Results in Differential Migrative and Invasive Capacities in Melanoma Cells

**Figure 3:**
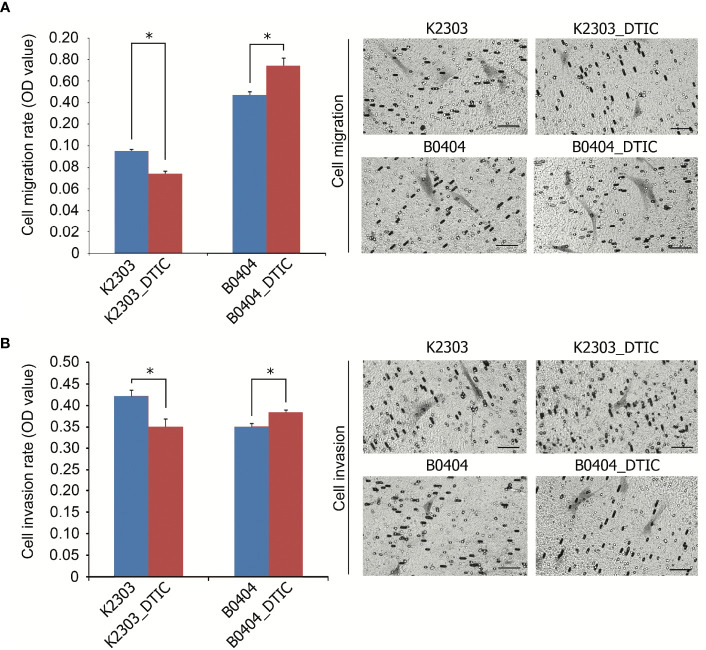
Invasive and migratory potential of K2303 and B0404 melanoma cells after DTIC treatment. **(A)** Migration assay results of cells that were treated with 5.5 mM DTIC. Microscopic images of the polycarbonate membrane with migrated cells are shown. Migratory capacities of K2303 cells were reduced whilst B0404 cells exhibited increased cell migration. Data are presented as the mean ± SEM. ^*^P<0.05 by Mann-Whitney U test for unpaired samples. DTIC, dacarbazine **(B)** Invasion assay results of cells that were treated with 5.5 mM DTIC. Microscopic images of the polycarbonate membrane with invasive cells are shown. Invasion rate of K2303 cells was reduced whilst the level of invasion of B0404 was increased. Data are presented as the mean ± SEM. ^*^P<0.05 by Mann-Whitney U test for unpaired samples. DTIC, dacarbazine.

The rates of migration and invasion were altered following DTIC treatment. We observed evident reductions in the migratory and invasive capacities of K2303 cells, whilst B0404 cells exhibited increased cell migration and invasion (**Figure 3**). Therefore, melanoma cells appeared to respond differentially in terms of the ability to disseminate in response to DTIC treatment in the present study.

### Transcriptomic Profile of Primary Melanoma Cells Following DTIC Treatment Is Characterized by Signaling Pathway Induction Associated With Cell Motility

**Figure 4:**
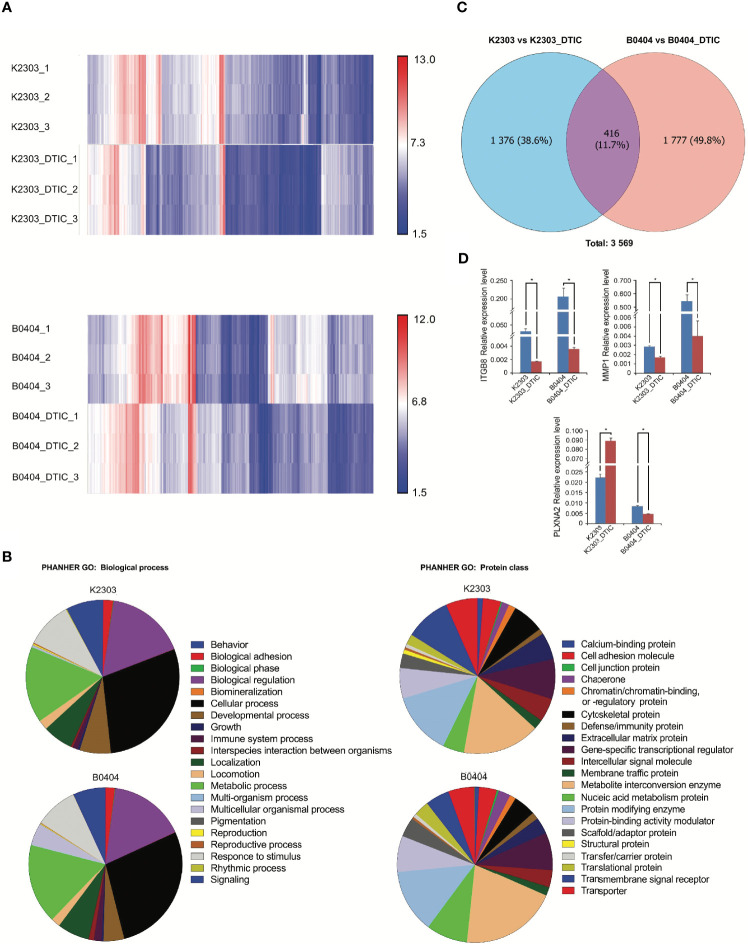
Results of whole transcriptome analysis of K2303 and B0404 melanoma cells. **(A)** Heatmaps showing differentially altered transcripts in K2303 and B0404 melanoma cells after DTIC treatment. **(B)** Gene Ontology annotations for the two sub-trees, representing the biological process and protein class, for genes with dysregulated expression after exposure to DTIC in K2303 and B0404 melanoma cells. Plots were made using the PANTHER™ v.16.0 classification system (http://www.pantherdb.org). **(C)** The Venn diagram shows the total number of altered transcripts in K2303 and B0404 melanoma cells after treatment with DTIC. **(D)** Changes in relative gene expression levels according to reverse transcription-quantitative polymerase chain reaction in K2303 and B0404 melanoma cells after 5.5 mM DTIC treatment. Data are presented as the mean ± SEM. ^*^P<0.05 by Mann-Whitney U test for unpaired samples. DTIC, dacarbazine.

To reveal the phenotype of primary melanoma cells obtained from the two different patients, we performed a transcriptomic study (**Figures 4A–C**). We identified a total of 5,042 genes with altered expression in K2303 cells and 8906 in B0404 cells after DTIC treatment. Upregulated genes in K2303 were generally associated with apoptosis (*TNFRS 10D*), DNA repair (*TDP2*), extracellular matrix remodeling (*TFPI2* and *RPTN*) and cell proliferation (*EPGN*, *GPRC5A*, *VASN*, *FOSB*, *SCUBE3* and *SULF1*). Among the list of downregulated genes, we observed them to be associated with cell proliferation (*FRAS1* and *ACAN)* and extracellular matrix remodeling *(ACAN)*. In B0404 cells, it was found that the genes that were upregulated following DTIC treatment were typically associated with T-cell mediated immune response (*GAGE1*), apoptosis and DNA repair (*RHOB*). By contrast, genes that were associated with cell proliferation (*AREG* and *DKK1*) and extracellular matrix remodeling (*MXRA5*, *MMP-1*, *EDIL3*, *CCDC80*, *TNC*, *ITGA8*, *FN1* and *CCBE1*) were downregulated.

We next focused our attention on signaling pathways where the differentially expressed genes were also components. We observed the top downregulated genes in B0404 to be components of the vascular endothelial growth factor A (VEGFA)/vascular endothelial growth factor receptor R2 (VEGFR2), PI3K/AKT, focal adhesion, interleukin-18, mitogen-activated protein kinase (MAPK), RAS and transforming growth factor (TGF)-β signaling pathways. Genes as components of PI3K/AKT, VEGFA/VEGFR2, focal adhesion, endothelin pathway, transforming growth factor (TGF)-β and hippo-Merlin signaling were downregulated in K2303 cells.

Since we found a clear dysregulation in the expression of genes associated with cell adhesion and migration, the expression levels of a number for these genes, components of up-regulated pathways were subsequently validated by RT-qPCR. Pathway Enrichment analysis was performed using the PANTHER database (http://pantherdb.org/). Gene Ontology (GO) terms associated with cancer cell biology (‘biological adhesion’, ‘biological regulation’, ‘cellular process’, ‘developmental process’, ‘localization’, ‘locomotion’, ‘multicellular organismal process’, ‘response to stimulus’ and ‘signaling’) were used to identify six genes matched to selected criteria, namely *ITGBL1*, *NOV*, *PLXNC1*, *ITGB3*, *ITGB8* and *PLXNA2*. Subsequently, *ITGB8* and *PLXNA2* were chosen as those most likely to be involved in the regulation of melanoma cell migration and invasion, as indicated in (22–24). *MMP1* was also selected as a key dysregulated gene in both cells following DTIC treatment, using the GO term ‘cellular process’. Thus, the mRNA levels of ITGB8, plexin-A2 (PLNXA2) and MMP1 were measured in both cell lines before and after DTIC treatment. ITGB8 and MMP1 downregulation were found in both cell lines. However, PLNXA2 mRNA expression was increased in K2303 cells, whereas PLNXA2 expression was reduced in B0404 cells after DTIC treatment (**Figure 4D**). The expression levels of all three of these genes corresponded accurately with the microarray data in the present study, although the MMP1 levels showed a tendency to decrease according to the microarray result but a significant decrease was observed in the RT-qPCR data. Thus, PLNXA2 down-regulation was associated with migrative and invasive phenotype of primary melanoma cells.

## Discussion

Recently, a number of approaches have been implemented for melanoma treatment to provide beneficial effects, including anti-programed death-1 antibodies, BRAF and MEK inhibitors (25). However, chemotherapeutic agents remain to be in use in clinical oncology. Cancer cells utilize a diverse of set of mechanisms to mediate resistance to chemotherapeutic agents based on alkylation, which explains why sensitivity can vary greatly (26). In the present study, we administered DTIC to primary melanoma cell lines obtained from two different patients. Before it, we analyzed cytochrome P1A1, cytochrome P1A2, and cytochrome P2E1 expression revealed by microarray and RT-qPCR in terms of ability to activate DTIC. We revealed cytochrome P1A1 expression in both cell lines, whereas cytochrome P1A2 expressed in B0404 cells, cytochrome P2E1 in K2303 cells. The cells exhibited response to DTIC in terms of the levels of apoptosis. Indeed, K2303 and B0404 melanoma cells responded by increasing the number of early apoptotic cells. In addition, B0404 cells demonstrated enhanced migratory and invasive capacities following DTIC treatment, which was opposite to that observed in K2303 cells, where reduced rates in both of these characteristics were observed. To unravel the possible underlying mechanism of this finding we performed a transcriptomic analysis in both of these cell lines before and after treatment with DTIC.

Transcriptomic analysis revealed that 416 genes were dysregulated significantly in both cell lines, which constituted only 12% of all differentially expressed genes with a fold change ≥2. Such modest uniformity is in the line with the phenotypic diversity observed in response to DTIC. DTIC was also found to trigger the expression of genes associated with DNA repair and apoptosis. Among those that were upregulated after being treated with DTIC were *RHOB*, *GCLM* and *TGM2*. In addition, a number of downregulated signaling pathways such as growth factor A (VEGFA)/vascular endothelial growth factor receptor R2 (VEGFR2), PI3K/AKT, focal adhesion were found to be associated with metastasis in both cell lines after treatment with DTIC, including motility, migration, invasion and adhesion, according to the transcriptomic profile analysis. This caught our attention as these aforementioned characteristics may affect the overall patient response and survival rates (27, 28). Therefore, the expression levels of a number of these chosen genes that were found to be associated with the aforementioned processes related to metastasis were measured by RT-qPCR. ITGB8 and MMP1 expression levels were reduced after DTIC incubation, which concurred with results from transcriptomic analysis. All three molecules have been previously shown to be implicated in tumor progression and metastasis of various cancer types including melanoma (22–24, 29, 30). ITGB8 is involved in TGF-β activation (31, 32), which was found to be downregulated after DTIC treatment in the present study. MMP1 is an important enzyme that is activated by TGF-β and mediates extracellular matrix degradation, especially those of collagen type I, II and III (33, 34). Therefore, ITGB8 and MMP1 may act unidirectionally following altered TGF-β signaling.

By contrast, we observed a reduction in PLXNA2 expression in B0404 cells but elevated PLXNA2 expression in K2303 cells, which is opposite to the trend observed in the migration and invasion assays following DTIC treatment. PLXNA2 belongs to the semaphorin family of proteins that is more synonymous with nervous system development (35, 36). However, PLXNA2 has been later reported to be implicated in the proliferation and invasion of breast cancer cells (37). Melanoma is originated from melanocytes, which are cells of neuronal origin. Therefore, genes in the semaphorin/plexin signaling pathway may serve a role in the biology of neoplastic melanocytes (18). Class A plexins function as receptors for classes 3, 5 and 6 of semaphorins (38). The signaling consequences of PLXNA2-semaphorins have been extensively studied in cancers of the nervous system (29, 39). Semaphorin 3C downregulation was reported to be associated with a more metastatic phenotype in neuroblastoma cells (40), consistent with our present findings of increased B0404 melanoma cell invasion and migration. In addition, recent study also showed that gene upregulation specific for embryonic melanoblasts is necessary to facilitate melanoma dissemination (41). The results of a previous study demonstrated that PLXNA2 played a key role in the regulation of perineural invasion of prostate cancer (42). Moreover, Tivan TV et al. demonstrated that migration and invasion were increased in prostate cancer cells treated with PLXNA2 small interfering RNA (30). The use of PLXNA2-knockout mice also revealed that PLXNA2 controls retinal cell migration during retinal neurogenesis (43). However, the underlying mechanisms by which this membrane-bound molecule is involved in the regulation of cell migration remain to be fully elucidated.

Transcriptomic profiling of tumors is under extensive assessment as a potential prognostic tool and as a method for predicting anti-cancer agent efficacy (44). However, the accuracy for the application of tumor-based gene expression analysis remains unverified and additional evidence is required prior to its use in routine clinical practice (45). Tumor heterogeneity may yet prove to be an obstacle to its clinical applicability, since the genomic landscape within any single melanoma tumor is characterized by significant differences. Melanoma remains a type of skin cancer with a high rate of cancer-associated deaths (46).

Nevertheless, this study has some limitations: the number of cell lines was limited due to obtaining of operative material from melanoma patients, efficacy of primary melanoma cells isolation for experiments *in vitro* and costs of transcriptomic study. Second, the microarray analysis was performed in three replicates that was also limited because of aforementioned reasons. The present study lacks PLXNA2-based mechanistic experiments due to difficulties in the cultivation and transfection of primary melanoma cells. However, all results discussed in this paper were reaching statistical significance level. The role of PLXNA2 in the regulation of melanoma cell invasion and migration may be a novel marker for the characterization of melanoma cells based on invasive or proliferative phenotypes. Such phenotypes may be used in predicting patient prognosis. Further studies should provide a more complete understanding of melanoma resistance to chemotherapeutic agents and cancer cell chemoresistance.

## Conclusions

Our findings suggest that other therapeutic options would need to be explored for melanoma. This is due to the high heterogeneity in the genetic landscape of the tumor and in the results from the functional study of cancer cells derived from individual patients. Data from the present study could be applied for further studies into phenotypic alterations in the tumor following the administration of anticancer agents.

## Data Availability Statement

The datasets presented in this study can be found in online repositories. The names of the repository/repositories and accession number(s) can be found in the article/supplementary material.

## Ethics Statement

The study was approved by the ethics committee of the Krasnoyarsk State Medical University (protocol no. 73/2016; approval date, 16 December, 2016) and the Krasnoyarsk Regional Clinical Oncology Center named after A.I. Kryzhanovskiy (protocol no. 8, 14, June, 2017). The patients/participants provided their written informed consent to participate in this study.

## Author Contributions

All authors contributed to the concept and design of the study. All authors contributed to the article and approved the submitted version.

## Funding

The study was supported by a grant from the Russian Science Foundation (project №19-15-00110).

## Conflict of Interest

The authors declare that the research was conducted in the absence of any commercial or financial relationships that could be construed as a potential conflict of interest.

## Publisher’s Note

All claims expressed in this article are solely those of the authors and do not necessarily represent those of their affiliated organizations, or those of the publisher, the editors and the reviewers. Any product that may be evaluated in this article, or claim that may be made by its manufacturer, is not guaranteed or endorsed by the publisher.
